# State of omics-based microbial diagnostics of CRC

**DOI:** 10.1080/19490976.2025.2526132

**Published:** 2025-07-02

**Authors:** Jerome Prusa, Mark G. Gorelik, Kevin S. Blake, Gautam Dantas

**Affiliations:** a Department of Pathology and Immunology, Division of Laboratory and Genomic Medicine, Washington University School of Medicine, St. Louis, MO, USA; b The Edison Family Center for Genome Sciences and Systems Biology, Washington University School of Medicine, St. Louis, MO, USA; c Department of Molecular Microbiology, Washington University School of Medicine, St. Louis, MO, USA; d Department of Biomedical Engineering, Washington University in St Louis, St. Louis, MO, USA; e Department of Pediatrics, Washington University School of Medicine, St. Louis, MO, USA

**Keywords:** Colorectal cancer, microbiome, microbial metabolites, diagnostics

## Abstract

Colorectal cancer (CRC) remains a major burden of cancer-related morbidity and mortality globally, especially when detected at later stages. Early detection through improved and more accessible diagnostics is critical for reducing the severity of CRC. As our understanding of CRC and the microbial inhabitants of the gastrointestinal tract continues to improve, it has become increasingly recognized that the bacterial component of the gut microbiome may provide diagnostic utility for detecting CRC. This is because CRC is often accompanied by shifts in bacterial taxa, and the metabolites produced or utilized by the CRC-associated gut bacterial community. Advances in sequencing and metabolite profiling technologies paired with our growing understanding of CRC-associated microbial taxa, present an opportunity for new gut microbiome-based diagnostics. In this narrative review, we discuss bacterial taxa and gut metabolites that have been investigated as predictive features for CRC diagnosis. We aim to highlight the tremendous progress that has been made in identifying gut microbiome-based features and why they should be further explored as potential CRC diagnostics. We also identify challenges that future work must address, including the impact of patient lifestyle, variation in methodology, and nonstandard data management practices. Resolving these areas of study design and implementation is key to understanding the complex bacterial communities and their by-products associated with CRC, and the development of microbial diagnostics that can detect them.

## CRC morbidity and mortality and importance of detection

Colorectal cancer (CRC) is the third most diagnosed cancer worldwide, and the second leading cause of cancer-related mortalities in the United States.^1^ Despite remaining one of the most prevalent and deadliest cancers, CRC incidence and mortality have been decreasing over the past several decades.^2^ This success has been attributed to increased screening for polyp removal and diagnosis of early-stage CRC, which has a 5-year survival rate of over 90%.^2,3^ However, screening can be costly and accessibility to the infrastructure required for certain methods (e.g. colonoscopies) can be limited. Furthermore, challenges to the continued progress in reducing the burden of CRC include the increased incidence and mortality among people under 50 years of age, the low 14% 5-year survival for late detected metastatic CRC, and only 59% of eligible persons 45 years of age or older adhering to screening guidelines.^4–7^ Improved, more accessible diagnostic approaches are critical for addressing these challenges. Importantly, recent advances in DNA sequencing technologies have led to decreased costs of sequencing and removed reliance on sequencing facilities.^8,9^ The rise of cheap sequencing methodologies has enabled exploration of microbial markers for measuring disease risk.

## Evidence that microbiome contributes to CRC induction

Several lines of evidence have indicated a role of the gut microbiome in CRC induction and tumorigenesis. Oral antibiotic use has been shown to increase the risk of CRC, presumably due to perturbations of the bacteria which reside in the colon.^10,11^ Similarly, dietary composition is also an important risk factor for CRC.^12,13^ In particular, low fiber intake, red meat, and alcohol consumption are all risk factors for CRC and have been shown to affect microbiome composition.^12,14^ In contrast, a high fiber diet is associated with a lower risk of CRC and similarly has been shown to impact the microbiome composition in a manner protective against CRC.^15^ In addition to lifestyle factors such as antibiotic usage and diet, studies investigating bacterial taxa enriched in the stool or tumor tissues of patients with colonic lesions have identified specific taxa that are reproducibly enriched. Some of these bacterial species associated with CRC include *Fusobacterium nucleatum*, *+pks Escherichia coli*, *Bacteroides fragilis*, *Peptostreptococcus stomatis*, and *Peptostreptococcus anaerobius*.^10,15–19^ Importantly, mechanistic studies investigating how these bacterial species might contribute to CRC tumorigenesis have identified gene products encoded by these taxa that directly promote CRC tumorigenesis.^13,20,21^

## Current CRC diagnostics

**Figure 1.**
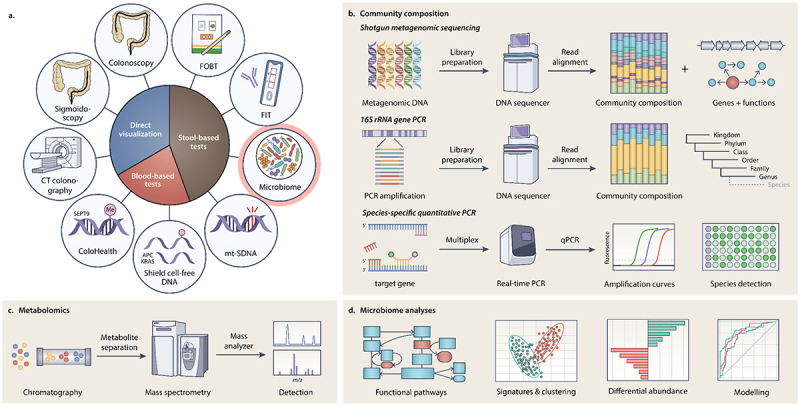
CRC diagnostic methodologies. a. Current routine screening and diagnostic approaches of CRC. b. Sequencing-based approaches for detecting taxa associated with CRC. c. Metabolomics-based approaches for detecting metabolites associated with CRC. D. Commonly used microbiome analysis for CRC based on -omics data.

Current CRC diagnostics include stool-based immunochemical tests, direct visualization, and blood-based tests.^7,22,23^ The stool-based tests include guaiac-based fecal occult blood tests (gFOBTs) and fecal immunochemical tests (FITs) that detect blood in the stool through either a pseudo peroxidase activity on the heme substrate or antibody detection of heme. A third stool-based test called the multi-target stool DNA (mt-SDNA) test detects abnormal CRC-associated host DNA in stool in conjunction with FIT-based blood detection (Figure 1(a)).^7,22–24^ Direct visualization screening methods include flexible sigmoidoscopy, colonoscopy, and computed tomography colonography (Figure 1(a)).^25^ Flexible sigmoidoscopy can visualize the distal bowel and colorectum, while the colonoscopy is able to visualize the entire colon. Computed tomography colonography (CTC) also allows direct visualization of the colon using CT scanning technology rather than a scope. Newer noninvasive diagnostics that detect CRC-associated DNA sequences from blood are also available, and include the ColoHealth assay that detects circulating methylated Sept9 DNA,^26^ and the Shield cell free DNA-based assay that detects a combination of aberrant methylation patterns, DNA fragmentation, and mutations in the KRAS and APC genes (Figure 1(a)).^27^

**Figure 2.**
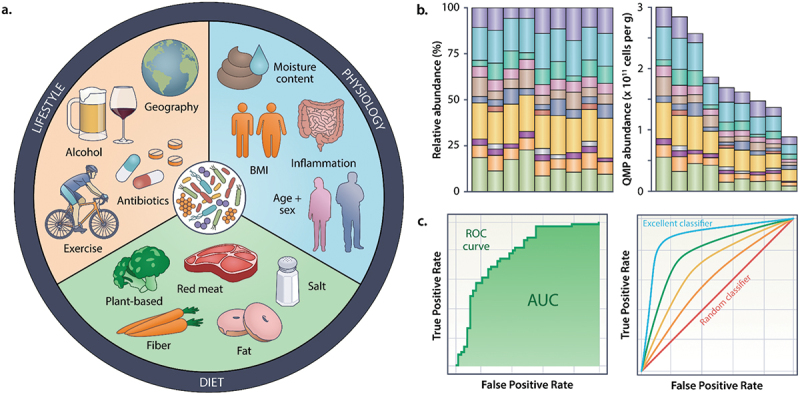
Caveats of microbiome analysis. A. Common factors which can impact microbiome composition and measurements. B. Implications of absolute vs. relative abundance C. Evaluating AUC results.

Collectively, these screening methods have reduced CRC incidences and mortalities and the efficacies of the screening methods have been rigorously reviewed and compared elsewhere.^25,28^ In addition to the currently approved diagnostics, ongoing clinical trials are evaluating diagnostics that seek to improve sensitivity and specificity for early-stage colonic lesion detection.^29^ Despite this progress, there are limitations to the currently approved diagnostics. Stool-based approaches are limited by low sensitivity, single sample usage, and requiring dietary and medication modifications (Figure 2(a)).^23,24^ Direct visualization methods have risk of complications,^22^ are more expensive,^30^ and are still susceptible to failures in identifying lesions.^10^ A promising avenue for improving stool-based diagnostics is utilizing microbially based markers premised by the abundant and growing evidence that colonic lesions are accompanied by discernable bacterial signatures within the gut microbiome (GM).^31,32^ Importantly, in addition to detecting the presence of CRC, bacterial signatures may provide additional insights into disease dynamics.

## Microbiome methodologies

Many studies investigating the diagnostic utility of GM-derived features leverage common methodologies with different implications. This brief summary aims to provide an overview of the approaches used in the sequencing studies described. These studies leverage one of three high-level approaches for determining community composition: species-specific quantitative PCR,16S rRNA gene PCR amplification and sequencing, and shotgun metagenomic sequencing (Figure 1(b)). Species-specific quantitative PCR is a probe-based approach that determines the presence and abundance of target species.^33–35^ While limited in breadth, species-specific probe-based approaches enable highly targeted quantification of specific taxa. Alternatively, studies which aim to capture high-level community composition often employ 16S rRNA PCR amplification-based approaches, which detect and quantify the abundance of bacterial taxa in a sample. The reduced taxonomic resolution of 16S sequencing is offset by increased breadth, as it can capture the overall taxonomic composition of a GM community rather than being limited to pre-selected probes.^36,37^ The third approach, shotgun metagenomic sequencing, overcomes the limitations of both species-specific PCR and 16S PCR, as it can capture overall community composition at the species, and potentially strain, level^38,39^ Additionally, since shotgun sequencing methods incorporate all genomic input material, they also capture data on encoded GM functions.^38^ The main drawback to shotgun sequencing is that it requires much greater sequencing depth, which is more expensive, as well as the current limitations of databases for shotgun and functional annotations.

Many microbiome studies have begun to generate and incorporate metabolomics data into their results^40–42^ (Figure 1(c)). By comparing the sequencing and metabolomics results of samples from healthy and diseased states, studies can identify which microbial taxa, pathways, and metabolites are most associated with these states. Linear modeling tools such as MaAsLin and ACNOMB-BC2 can be used to identify taxa which are differentially abundant in healthy or disease associated samples.^43,44^ Clustering approaches, often utilizing principal component analysis, can be used to determine if the overall microbiome composition differs by disease state. In the case of shotgun sequencing, mapping reads to functional pathway databases can be used to determine if pathways are differentially abundant between states, and metabolomics data can be used to identify small molecules produced by the relevant biosynthetic pathways. Finally, modeling approaches are often used to determine the diagnostic utility of taxonomic, functional, and metabolite features identified in the aforementioned analyses. GM-derived features can be added to pre-existing models to see if this improves performance, or models can be trained and evaluated using only GM-derived measurements (Figure 1(d)).

## Increased abundance of oral anaerobes

The best characterized bacterial feature distinguishing the gut microbiomes of CRC patients from healthy individuals is the increased abundance of a group of oral anaerobes in the colonic mucosa and stool of CRC patients.^21,45^ These oral anaerobes include *F. nucleatum*, *P. micra*, *P. stomatis*, *P. anaerobius*, and *Porphyromonas asaccharolytica* among other species from the *Fusobacterium* and *Porphyromonas* genera.^16–18,21^ The diagnostic utility of measuring these species individually or in various combinations with one another or other bacterial taxa has been studied extensively with varying molecular methods including directed quantitative PCR, marker-based amplicon 16S rRNA sequencing, and shotgun metagenomic sequencing (Figure 1(b)).^16,35,46–48^

Among the oral anaerobes, *F. nucleatum* has been studied the most extensively as a mechanistic contributor to CRC pathogenesis. Mechanisms that have been described for *F. nucleatum* include direct induction of CRC proliferation and metabolic reprogramming, stimulating a pro-inflammatory CRC tumor microenvironment, inhibition of anti-tumor immune responses, increasing genetic and epigenetic lesions, and promoting both CRC metastasis and chemoresistance. The bacterial and host cell effectors, signaling pathways, and other molecular details underpinning *F. nucleatum*’s various roles in CRC pathogenesis have been reviewed extensively.^21,45,49,50^

Many studies have examined the predictive power of *F. nucleatum* abundances in the stool or colonic mucosa for distinguishing healthy individuals from those with varying stages of CRC neoplasms. This large body of work addressing this question has allowed for multiple secondary meta-analyses that have evaluated the results from over 50 studies and are highlighted below.

The diagnostic accuracy for distinguishing between CRC cases and healthy individuals using *F. nucleatum* or other microbial biomarkers is quantified with two measurements: 1) the area under the receiver operating characteristic curve (AUROC) that reports an AUC value and 2) the diagnostic odd-ratio (DOR).^18,46,51^ The AUC is measured from plotting the receiver operating characteristic (ROC) curve on a two-dimensional plot with the false-positive rate on the x-axis (1 - specificity) and the true positive rate (sensitivity) on the y-axis. A curve closer to the upper left-hand corner indicates higher prediction accuracy with a maximum value of 1.0. AUC values must have a value of greater than 0.5 to have any diagnostic value. Values between 0.5 and 0.75 are considered poorly diagnostic, values between 0.75 and 0.9 as acceptable, and values above 0.9 as excellent (Figures 1d, 2c).^51^ The DOR is the ratio of true positives to false positives, thus a value higher than one indicates higher diagnostic accuracy.^52^

The meta-analysis by Peng et al.^46^ included a total of 1198 participants (629 individuals with CRC and 569 healthy controls) from 10 studies published in 7 peer-reviewed articles and evaluated the classification accuracy from the presence of *F. nucleatum* for classifying healthy individuals from CRC cases. This analysis measured an AUC = 0.86 (95% CI: 0.83–0.89) and DOR = 14.0 (95% CI: 9.00–22.0). Another meta-analysis reported by Huang et al. included seven case-control studies, published in six peer-reviewed articles with a total of 557 CRC patients, and 704 healthy controls, where an AUC = 0.8 was observed, and a DOR = 8.75 (95% CI: 8.75) using the presence of *F. nucleatum* in stool for classifying healthy controls vs. CRC patients.^18^ Zhang et al. also reported an AUC = 0.8 (95% CI: 0.76–0.83) using the presence of *F. nucleatum* in stool for classifying healthy individuals from CRC patients, while also reporting an AUC = 0.6 (95% CI: 0.56–0.65) for classifying healthy individuals from patients with precancerous colorectal adenomas (CRA) using pooled data from 10 published articles including a total of 1450 CRC patients and 1421 controls.^53^ Gethings-Behncke et al. evaluated data from a total of 45 published articles and reported a DOR of 10.6 (95% CI:4.48–22.58) for classifying healthy individuals from those with colorectal polyps using the presence of *F. nucleatum* in colorectal tissues and a similar DOR = 9.01 (95% CI: 3.39–23.95) using *F. nucleatum* detection in the stool.^15^ These meta-analyses that collectively evaluated the utility of *F. nucleatum* detection for CRC classification from the numerous studies suggest that *F. nucleatum* is an acceptable microbial-based feature for CRC detection.

Despite the numerous studies suggesting that *F. nucleatum* could serve as a valuable diagnostic marker, a recent study evaluating stool 16S rRNA profiles of a large 589 CRC patient cohort along with a meta-analysis including 15 published studies with a total of 4,439 CRC patients and healthy controls determined that *F. nucleatum* was not significantly associated with the diagnostic groups included in the analysis.^54^ This discrepancy between this study and the other studies that had identified *F. nucleatum* as powerful differentiating feature was attributed to this study rigorously controlling for confounding factors such as body mass index, calprotectin levels in the stool, and transit time (water content measured as a proxy for transit time) and the use of quantitative 16S rRNA measurements determined by the inclusion of a spike-in feature rather than through relative microbiome profiling that has been used in the studies reporting *F. nucleatum* as a CRC-associated taxa. This study raises questions around the status of *F. nucleatum* as a potential CRC diagnostic and highlights the importance of controlling for potential confounding variables and the limitations of relative abundance measurements from 16S rRNA microbiome profiling approaches. However, other CRC-associated oral anaerobes including *P. micra*, *P. anaerobius*, and *P. asaccharolytica* were validated as predictive CRC features in this study.^54^

Compared to *F. nucleatum*, far fewer studies have examined how well marker-based qPCR abundance measurements of *P. micra*, *P. anaerobius, P. stomatis*, or *P. asaccharolytica* in stool or colonic lesions can individually differentiate healthy individuals from CRC patients. For *P. micra*, at least three studies have reported the ability to differentiate CRC patients from healthy individuals with qPCR measurements of *P. micra* abundances. Two studies have reported very similar AUC values, including 0.73 reported in Wong et al.,^55^ and 0.726 in Lowenmark et al.,^56^ while Osman et al. reported *P. micra* having an AUROC = 0.908.^33^ Wong et al. also reported the AUC for differentiating healthy individuals from CRC patients using qPCR measurements of *P. anaerobius* and report a value of 0.72,^55^ while Osman et al. included *P. stomatis* among the group of 5 species that AUROC values were measured and reported a value of 0.795.^33^ The results from these studies indicate that these less-characterized oral anaerobes have promising predictive value for CRC diagnostics, but the utility of a single species marker may be limited.

The potentially limited ability to accurately differentiate healthy individuals from CRC patients with a single bacterial species, even when utilizing the species thought to be most strongly associated with CRC, can be improved through multi-taxa microbial panels. Several studies have identified and utilized these panels and are highlighted in Table 1. These studies share a similar design, including a first step of analyzing a metagenomic dataset or datasets generated from stool samples collected from either individuals with colorectal lesions or healthy controls and identifying the microbial taxa that are most strongly associated with either group. The multi-marker panels are then evaluated for their ability to classify healthy individuals from CRC patients within the datasets that were used to discover the discriminating features or external independent datasets.^19,24,41,42,47,48,55,57–60^

**Table 1.** Bacterial taxa used for CRC detection.

Study	Feature Discovery and Validation Cohorts	Bacterial Features used for classification	AUROC
Yu et al.^30^	5 cohorts included − 1 new metagenomic dataset (healthy controls, *n* = 54; CRC patients, *n* = 74) used for feature discovery. Feature panel was evaluated in 3 publicly available CRC metagenomic cohorts (healthy controls, *n* = 140; CRC patients, *n* = 110) and qPCR validated in stool samples (healthy controls, *n* = 109; CRC patients, *n* = 47)	4 microbial gene markers (2 from *Peptostreptococcus anaerobius*, 1 from *Parvimonas micra* and 1 from *Fusobacterium nucleatum*) used for AUC classification of healthy vs CRC patients in two metagenomic datasets. 2 microbial gene markers (butyryl-CoA dehydrogenase from *Fusobacterium nucleatum* and rpoB from *Parvimonas micra*) and measured by qPCR from stool samples used for AUC classification of healthy vs. CRC.	Healthy vs CRC classification AUC = 0.77 and 0.72 discriminated by 4 bacterial gene markers in two metagenomic datasets. AUC = 0.84 using 2 bacterial gene markers measured by qPCR in a single cohort of stool samples.
Dai et al.^75^	4 publicly available CRC metagenomic datasets used for feature discovery and AUC measurement (healthy controls, *n* = 271; CRC cases, *n* = 255).	7 bacterial species panel for AUC classification of healthy vs. CRC patients - *Bacteroides fragilis, Fusobacterium nucleatum, Porphyromonas assacharolytica, Parvimonas micra, Prevotella intermedia, Alistipes finegoldii, Thermanaerovibrio acidaminovorans*	Healthy vs. CRC classification AUC = 0.80 across the combined population from the 4 metagenomic datasets using the 7 bacterial species panel.
Wirbel et al.^57^	8 cohorts included − 1 new metagenomic dataset (healthy controls, *n* = 60; cancer cases, *n* = 60) and 4 additional publicly available CRC metagenomic datasets (healthy controls, *n* = 230; cancer cases, *n* = 225) used for feature discovery. Feature panel was evaluated in the 5 feature discovery datasets and 3 additional publicly available metagenomic CRC external cohorts (healthy controls, *n* = 92; cancer cases, *n* = 101)	29 bacterial species panel for AUC classification of healthy vs. CRC patients – top 9 effect-size features are *Parvimonas micra, Gemella morbillorum*, *Peptostreptococcus stomatis*, *Fusobacterium nucleatum* ssp. *animalis*, unknown *Dialister* sp., unkown *Porphyromonas* sp., *Solobacterium moorei*, *Porphyromonas uenonis, Clostridium symbiosum*	Healthy vs CRC classification AUC range of 0.71–0.91 by LOSO analysis across the 5 cohorts used for feature discovery and healthy vs CRC classification AUC range 0.79–0.81 across the 3 external validation cohorts using the 29 bacterial species panel.
Thomas et al.^61^	7 cohorts included − 2 new metagenomic datasets, cohort 1 (healthy controls, *n* = 24; CRA, *n* = 27; CRC, *n* = 29), cohort 2 (healthy controls, *n* = 28; CRC, *n* = 32) and 5 publicly available CRC metagenomic datasets (healthy controls, *n* = 256; CRA, *n* = 116; CRC, *n* = 252). Feature panel was evaluated for all 7 cohorts.	16 bacterial species panel for AUC classification of healthy vs. CRC patients – top ranked species included in the panel are *Peptostreptococcus stomatis*, *Fusobacterium nucleatum*, *Parvimonas* spp., P*orphyromonas asaccharolytica*, *Gemella morbillorum*, *Streptococcus salivarius*, *Clostridium symbosium*, *Eubacterium eligens*, *Parvimonas micra*	Healthy vs. CRC average classification AUC > 0.8 across the 7 cohorts by LOSO analysis using the 16 bacterial species panel. Healthy vs CRC classification AUC = 0.91 and 0.77 in 2 external validation cohorts using the 16 bacterial species panel.
Yachida et al.^58^	Single new metagenomic dataset including a total of 616 people (healthy controls, *n* = 251; multiple polypoid adenomas with low grade dysplasia (MP), *n* = 67; stage 0, *n* = 73; stage I/II, *n* = 111; stage III/IV, *n* = 74; individuals with a history of colorectal surgery (HS), *n* = 40) used for feature discovery and AUC measurements.	55 bacterial species panel used for AUC classification of healthy vs SIII/IV CRC patients – top features include *Fusobacterium. nucleatum* ssp. *nucleatum*, *Parvimonas micra, Peptostreptoccus stomatis, Fusocbacterium nucleatum* ssp. *vincentii*, *Peptostreptococcus anaerobius*. 29 bacterial species panel used for AUC classification of healthy vs S0 CRC patients – top features include *Bacteroides bamesiae*, *Bacteroides coprophilus*, *Desulfovibrio longreachensis*, *Bacteroides salanitronis*, *Bacteroides plebius*	Healthy vs SIII/IV CRC classification AUC = 0.83 using a 55 bacterial species panel. Healthy vs S0 CRC classification AUC = 0.73 using a 29 bacterial species panel.
Liu et al.^59^	8 cohorts included − 1 new metagenomic dataset (healthy controls, *n* = 86; CRC, *n* = 80). Feature panel was identified with five publicly available CRC metagenomic datasets (healthy, *n* = 494; CRC, *n* = 491), Feature panel was evaluated in all 5 discovery cohorts and 3 additional metagenomic CRC datasets including the new metagenomic dataset (healthy, *n* = 190; CRC, *n* = 193).	11 bacterial species among the 16-feature panel used for AUC classification of healthy vs CRC patients – *Gemella morbillorum*, *Parvimonas micra*, *Ruminococcus biriculans*, *Roseburia instestinalis*, *Fusobacterium nucleatum*, *Pseudobutyrivibrio xylanivorans*, *Streptoccocus anginosus*, *Eubacterium eligens*, *Faecalibacterium prausnitzii*, *Cellulosilyticum* sp. WCF-2, *Mogibacterium diversum*	Healthy vs. CRC classification average AUC = 0.83 for the 5 cohorts used for feature discovery, and AUC > 0.8 for 2 of 3 external validation cohorts using the 16 microbial feature panel.
Gao et al.^41^	8 cohorts included − 1 new metagenomic dataset (healthy controls, *n* = 91; CRC, *n* = 71; CRA, *n* = 63) used for feature discovery. Feature panel was evaluated in 7 additional publicly available CRC metagenomic datasets (healthy, *n* = 429; CRC, *n* = 431)	12 bacterial species panel used for AUC classification of healthy individuals vs. CRC patients (ordered by importance in panel) – *Parvimonas* unclassified, *Fusobacterium nucleatum*, *Roseburia intestinalis*, *Gardnerella vaginalis*, *Peptostreptococcus anaerobius*, *Peptostreptococcus stomatis*, *Lactobacillus iners*, *Gemella morbillorum*, *Porphyromonas asaccharolytica*, *Faecalibacterium prausnitzii*, *Atopobium vaginae*, *Parvimonas micra*	Healthy vs. CRC classification AUC range of 0.727–0.869 across the 7 cross-validation cohorts using the 12 bacterial species panel.
Coker et al.^47^	One new metagenomic dataset including a total of 386 people used for feature discovery and AUC measurement (healthy controls, *n* = 128; CRA, *n* = 140; CRC, *n* = 118)	6 bacterial species panel used for AUC classification of healthy individuals vs CRC patients – *Fusobacterium nucleatum*, *Peptostreptococcus anaerobius*, *Parvimonas micra*, *Roseburia inulinivorans*, *Eikenella corrodens*, and *Xanthomonas perforans*. 14 bacterial species panel used for AUC classification of healthy individuals vs CRA patients – *Eubacterium cellulosolvens*, *Lachnospiriceae* spp., *Clostridium bolteae*, *Streptococcus tigurinus*, *Xanthomonas gardneri*, *Eikenella corrodens*, *Oscillibacter valericigenes*, *Actinomyces viscosus*, *Clostridium symbiosum*, *Prevotella intermedia*, *Slackia exigua*, *Prevotella nigrescens*, *Porphyromonas gingivalis*, *Synergistes* spp.	Healthy vs. CRC classification AUC = 90.5 using the 6 bacterial species panel. Healthy vs. CRA classification AUC = 84.08
Avuthu et al.^60^	3 publicly available CRC metagenomic datasets used for feature discovery. Feature panel was evaluated is the 3 discovery cohorts (healthy controls, *n* = 166; CRC, *n* = 180) and 2 additional publicly available CRC metagenomic validation datasets (healthy controls, *n* = 303; CRC, *n* = 304).	21 bacterial species panel used for AUC classification of healthy individuals vs CRC patients – top 6 size-effect CRC enriched bacterial species include *Clostridium symbiosum*, *Fusobacterium nucleatum*, *Ruminococcus torques*, *Gemella morbillorum*, *Solobacterium morbillorum*, *Parvimonas micra*. Top 6 size-effect CRC depleted bacterial species include *Eubacterium eligens*, *Eubacterium ventriosum*, *Coprococcus* spp., *Aldercreutzia equalifaciens*, *Burkholderiales* spp., *Eubacterium halli*	Healthy vs. CRC classification AUC average range 0.62–0.78 for the 3 cohorts used for feature discovery and AUC = 0.66 and 0.61 for the 2 external validation cohorts using the 21 bacterial species panel.
Kong et al.^42^	Total of 2 cohorts included − 1 new metagenomic dataset for feature discovery including a total of 441 people (healthy controls for late onset (LO) CRC (>50 years of age), *n* = 97; LO-CRC, *n* = 130; healthy controls for early onset (EO) CRC (<50 years of age), *n* = 100; EO-CRC, *n* = 114). One new metagenomic dataset for feature validation including a total of 108 people (healthy controls for LO-CRC, *n* = 22; LO-CRC, *n* = 38; healthy controls for EO-CRC, *n* = 24; EO-CRC, *n* = 24.	32 bacterial species panel used for AUC classification of LO-CRC and LO-control – top 6 differentially abundant species are *Cellulosilyticum* spp., *Fusobactrium nucleatum*, *Anaerostipes hadrus*, *Lachnoclostridium phytofermentans*, *Roseburia intestinalis*. 49 bacterial species panel used for AUC classification of EO-CRC vs. EO-CRC control – top 4 differentially abundant species are *Faecalibacterium prausnitzii*, *Roseburia intestinalis*, *Lachnospirichiae* spp., *Eubacterium rectale*	LO-CRC vs. LO control AUC = 0.7817 for validation cohort using the 32 bacterial species panel and EO-CRC vs. EO control AUC = 0.7734 for validation cohort using the 49 bacterial species panel.

These feature discovery and validation studies lend further support that oral anaerobes can collectively be used to accurately detect neoplastic colonic lesions as demonstrated by *F. nucleatum*, *P. micra*, *P. stomatis*, *P. anaerobius*, and *P. asaccharlytica* being consistently enriched among CRC patients and used as one of the key features for classifier models in several of these studies. In addition to these well-established CRC-associated oral anaerobes, additional oral and gut commensals were also repeatedly identified as enriched among patients with colonic lesions. These taxa include *Gemella morbillorum*, *Solobacterium moorei* and *Clostridium symbiosum* .^47,57–61^

*G. morbillorum* is a facultative anaerobic gram-positive Bacillota cocci and a commensal of various mucosal surfaces including both the mouth and the gut.^62^ Importantly, *G. morbillorum* can cause opportunistic infections, most importantly endocarditis.^63^ In addition to the studies highlighted in Table 1, additional studies have also shown that *G. morbillorum* is enriched in the stool of CRC patients relative to healthy controls,^16,35^ and patients hospitalized with *G. morbillorum* bacteremia are at a higher risk of CRC.^48^ Whether the abundance of *G.morbillorum* can accurately classify CRC patients from healthy controls was evaluated in two cohorts with a total of 439 individuals in Yao et al..^64^ The AUC measured from this study, 0.799 (95% CI: 0.751–0.842) suggests that *G. morbillorum* is a viable marker for indication of colonic lesions and is worth further exploration into the mechanism of *G. morbillorum* association with CRC and utility of *G. morbillorum* for colonic lesion detection.

Similarly, *S. moorei* is a facultative anaerobic gram-positive Bacillota commensal of the gut microbiome but has been best characterized as an oral pathogen responsible for halitosis.^65^ The periodontal pathogenesis of *S. moorei* is impart due to *S. moorei* production of volatile sulfur compounds, such as hydrogen sulfide. Hydrogen sulfide is genotoxic and an enrichment of hydrogen sulfide producing taxa could directly contribute to colonic tumorigenesis.^66^ In addition to the production of hydrogen sulfide, *S. moorei* attachment to colonic epithelium stimulates epithelial proliferation and the production of pro-inflammatory effectors including diamine oxidase, D-lactate, C-reactive protein, TNF-alpha, IL-6 and IL-1-beta, while also suppressing the proliferation of CD4+ and CD8+ T cells.^67^ To our knowledge the utility of using *S. moorei* as a feature for classifying CRC has not been determined, and future studies investigating additional CRC predictive taxa will hopefully elucidate the diagnostic utility of *S. moorei*.

*Clostridium* spp. have been implicated in CRC pathogenesis, but the role this taxa plays is complex with a mixture of beneficial and detrimental mechanisms reported and ambiguity as to whether enrichment or depletion of *Clostridium* spp. occur in CRC patients. The best described protective role of *Clostridium* spp. is the production of short chain fatty acids (SCFAs), most notably butyrate, and the SCFA-dependent and independent roles in maintaining the integrity of the gut epithelium, modulating the pro- and anti-inflammatory pathways including Th17, Treg, and CD4/CD8 T cell proliferation, NF-kappa-B signaling, and inhibition of the Wnt/beta-catenin signaling cascade.^68^ However, *Clostridium* spp. have also been shown to promote CRC tumorigenesis via several different molecular mechanisms, some of which involve the production of SCFAs, demonstrating the complex balance of microbial metabolites required for gut homeostasis and the apparent context dependent role that SCFAs play in CRC pathogenesis.^69^ *C. symbiosum* was identified as one of the most enriched species among CRA and CRC patients among several of the large metagenomic studies highlighted in Table 1 and included in the panels of taxa accurately differentiating healthy individuals from patients with colorectal lesions.^47,57,60,70^ In addition to these multi-cohort feature discovery reports, the utility of *C. symbiosum* for CRA or CRC detection has been evaluated by qPCR quantification in a cohort including 212 CRA and 109 CRC patients with tumors restricted to the colonic submucosa, classified as early CRC, 218 patients with advanced CRC, and 242 healthy controls.^71^ Xie et al. report that the abundance of *C. symbiosum* outperformed the other predictive markers evaluated including relative abundances of *F. nucleatum* and the fecal immunohistochemical test (FIT), and the combination of *C. symbiosum* relative abundances with FIT was able to achieve an AUC = 0.803 for early CRC classification. How *C. symbiosum* promotes CRC pathogenesis is beginning to be elucidated with a recent study showing that cholesterol synthesis stimulated by *C. symbiosum* produced SCFAs induce CRC tumorigenesis via the Sonic hedgehog signaling pathway.^69^ The data suggest that *C. symbiosum* can contribute to CRC pathogenesis and provides strong rationale for continued investigation of *C. symbiosum* as a prognostic for colonic lesions with further studies.

Numerous other bacterial species have been associated with CRC tumorigenesis and to varying extents have been explored as diagnostic markers. Two broad categories that include many of these taxa are 1) species that are sometimes referred to as “driver” species according to the “driver-passenger” hypothesis^48,72–74^ and 2) healthy gut-associated commensals thought to play a protective role against colonic lesions which are lost during tumorigenesis.^42,59,75^ Well-studied CRC-associated taxa including *pks*+ *E. coli*, *B. fragilis*, and *F. nucleatum* are sometimes referred to as “driver” species due to their production of effectors such as colibactin, *B. fragilis* toxin (BFT), and *Fusobacterium nucleatum*’s FadA and Fap2, that directly promote CRC tumorigenesis through DNA damage or inducing oncogenic signaling pathways.^21^ In addition to these taxa, other bacterial species that have been investigated as CRC drivers are *Streptococcus gallolyticus*, *Salmonella enterica* as well as the oral anaerobes discussed above including the *Porphyromonas* species, *P. anaerobius*, and *P. micra*, which to varying extents have been evaluated as diagnostic features.^20,21,33,56,73,74,76^ The evidence supporting these taxa functioning as CRC drivers are both epidemiological data showing their increased abundances in CRC patients compared to healthy individuals and mouse data showing that at varying dosing regimens these taxa induce colonic tumorigenesis. Several excellent reviews have highlighted the discovery and characterization of CRC driver species and underscore the large amount of evidence that these taxa contribute to CRC.^20,21,73,74^ However, the utility of these important driver species for detecting neoplastic lesions is variable. While some driver taxa, such as the oral anaerobes, most notably *F. nucleatum* have been explored and continue to be investigated as diagnostic markers, there is evidence that driver species are replaced in the gut community by the so-called CRC passenger taxa making the potential use of the driver taxa as diagnostics more challenging.^72^ This is supported by the findings across the large cohort meta-analyses summarized in Table 1, where *B. fragilis* and *E. coli* are noticeably absent among the top features included in the classification models. Conversely, the second group of taxa, healthy gut-associated commensals, and their CRC-associated depletion serving as a predictive marker for CRA or CRC detection is supported by several of the meta-analyses highlighted in Table 1.^41,47,58,61,75^ The healthy gut commensal taxa that were depleted and included as taxonomic features for classification models include some of the most prominent SCFA producing *Clostridiales* commensals including *F. prausnitzii*, *Roseburia intestinalis*, and *Eubacterium* spp.

Depletion of *Clostridiales* species was among one of the first CRC-associated markers recognized in studies comparing the fecal microbiomes of CRC patients and healthy individuals.^77,78^ Perhaps the best characterized commensal consistently depleted among CRC patients across multiple studies is *F. prausnitzii*.^34,79–81^ Members of the *Faecalibacterium* genus serve as a central node for carbohydrate metabolism and cross feeding important for gut homeostasis.^82^ In addition to the studies highlighted in Table 1, other studies have evaluated *F. prausnitzii* as a predictive feature for distinguishing CRC patients from healthy controls. In Cao et al. *F. prausnitzii* was one three bacterial taxa (also including *R. intestinalis*, and *F. nucleatum*) included among a 15 feature panel along with a panel of T cell receptor beta sequences found to be enriched in CRCs that was able to differentiate healthy individuals from stage I and II CRC patients as well as healthy controls from stage III and IV CRC patients with a high accuracy at an AUC = 0.975 and AUC = 0.9725, respectively, in a validation cohort of 26 CRC patients and 20 healthy individuals.^83^ In another recent study investigating bacterial shifts associated with overweight and obesity-related CRC, *F. prausnitzii* was among the top features of a 48 bacterial taxa panel that classified healthy individuals from overweight-associated CRC patients and obesity-associated CRC patients with an AUC range of 0.7374–0.9778.^84^ The molecular mechanisms that underpin the protective role that the prominent SCFA producers such as *F. prausntizii*, *R. intestinalis*, and *Eubacterium* spp. for preventing CRC and other GI-related diseases are actively being investigated.

## Microbial-derived metabolites for CRC diagnostics

In addition to efforts to identify bacterial taxa that differentiate healthy individuals from those with colonic lesions, the metabolites produced by the differentially abundant taxa have been an active area of discovery (Figure 1(c)).^47,85,86^ Some of the metabolites that have been explored as CRC diagnostics include secondary bile acids, trimethylamine, short-chain fatty acids, indoles, polyamines, and various amino acids. Similar to the bacterial taxa shown to drive tumorigenesis, the molecular mechanisms that allow these well-studied metabolites to promote tumorigenesis are expansive and have been detailed elsewhere.^47,85^ Below we highlight some of the studies that evaluated the diagnostic utility of these metabolites associated with CRC.

## Bile acids

Bile acids are cholesterol-derived metabolites with detergent-like properties that are produced in the liver and promote the emulsification and absorption of fat-soluble molecules in the small bowel.^87^ The majority of bile acids (95%) are reabsorbed in the small bowel, however the fraction not reabsorbed are transformed via deconjugation, dehydroxylation, epimerization and oxidation by microbes, including a number of gram-positive genera such as *Clostridium*, *Lactobacillus*, *Enterococcus*, and *Bifidobacterium* as well as certain *Bacteroides*.^88,89^ The primary bile acid transformations produce a diverse pool of secondary bile acids.^90^ Though there are many secondary bile acids, much of the research focused on the role of bile acids in CRC has focused on the secondary bile acid deoxycholic acid derived from the primary bile acid cholic acid and the secondary bile acid lithocholic acid derived from the primary bile acid chenodeoxycholic acid.^91^ How secondary bile acids promote CRC tumorigenesis is multifaceted and several mechanisms have been described and reviewed elsewhere.^92^ These mechanisms include damaging the colonic epithelium due to the detergent-like properties of bile acids which in turn causes increased proliferation of undifferentiated cells and repair mechanisms mediated by inflammatory pathways. Bile acids also directly damage DNA and other macromolecules through stimulation of reactive oxygen and nitrogen species, resulting in genomic instability. Bile acids have also been shown to block apoptosis of gut epithelial cells through degradation of p53 and inhibition of caspase-3 mediated apoptosis, while also modulating oncogenic signaling pathways Wnt/beta-catenin and NF-kappa-B, to promote CRC tumorigenesis through altering epithelial proliferation, differentiation, growth, and apoptosis. More recent work has shown that secondary bile acids can also impact the immune system’s anti-tumor mechanisms.^93,94^ The in-depth and ongoing characterization of bile acid’s toxic and tumorigenic impact on host colonic epithelia as well as other cell types suggests that bile acids would likely be found at higher levels in biospecimens collected from cancer patients.^91,92^ This question has been investigated in numerous observational studies comparing fecal bile acid levels between CRC or CRA patients and healthy controls to identify differentially abundant primary or secondary bile acids. These studies have yielded varying results across studies, however the majority report that one or more bile acid variants are found at higher levels in the CRA and CRC patient stools compared to healthy controls. However, a meta-analysis including 19 of these observational studies conducted between 1975 and 2005 only partially supports this notion, where the primary bile acid chenodeoxycholic acid was the single bile acid found at significantly higher levels in the stools from CRA and CRC patients, while secondary bile acids were found to not be differentially abundant.^95^

More recent studies have continued investigating whether fecal bile acid levels are altered in patients with colonic lesions utilizing large metagenomic and metabolomic datasets and collectively have strengthened the case that bile acid producing taxa along with the bile acids they produce are implicated in CRC tumorigenesis and could potentially be leveraged as a diagnostic marker for colonic lesions. In Wirbel et al. the *bai* operon encoded by *Clostridiales* species, which encodes the gene products allowing primary to secondary bile acid conversions, was enriched in CRC metagenomes across all five datasets included in the study. Validation and ability to differentiate between CRC and healthy metagenomes were evaluated with qPCR measurements of the *baiF* gene within the *bai* operon, which as an individual feature yielded an AUC = 0.77 from a validation cohort of 47 fecal samples.^57^ Metabolomics analysis in Kong et al. also identified cholic acid and deoxycholic acid at significantly higher levels in the fecal samples of 114 early onset CRC patients compared to 100 age matched controls, while metagenomic analyses in this same study found enrichment of *cbh*, an enzyme involved in cholic acid to deoxycholic acid conversion in the metagenomes derived from early onset CRC patients.^96^ Likewise, Yachida et al. that included a metabolomic analysis of 409 stool samples from patients with colonic lesions of varying severity ranging from multiple polypoid adenomas (MP) to stage IV CRC, identified significantly increased levels of deoxycholic acid in fecal samples from MP patients and significantly increased levels of primary bile acids, glycocholate and taurocholate in stage 0 CRC patients compared to healthy controls, suggesting that altered bile acid levels are a valuable indicator for early stage colonic lesions.^58^ Another large metabolomics comparison of stool samples from CRA patients (*n* = 102) and healthy individuals (*n* = 102), also found higher levels of 3-beta-hydroxy-5-cholenoic acid and deoxycholic acid in CRA patient stool samples.^97^ An interesting trend across these studies is bile acid enrichment among patients with early-stage colonic lesions. These results may indicate that increased bile acid metabolism occurs early during tumorigenesis and could be best utilized in a stool-based diagnostic that differentiates healthy individuals from patients with pre-cancerous lesions.

An alternative to a fecal-based bile acid CRC or CRA diagnostic is a serum-based assay. Most bile acids are reabsorbed through active transport in the distal ileum or passive transport throughout the intestine and circulated back to the liver via the hepatic portal vein; however, 10–30% of bile acids escape liver retraction, circulate systemically, and can be quantified in serum.^98^ Two studies have shown that several primary and secondary bile acids positively correlated with CRC risk. The 7 bile acids identified in Kuhn et al. include the primary bile acids 1) glycocholic acid (DOR = 2.22: 95% CI = 1.52–3.26), 2) taurocholic acid (DOR = 1.78: 95% CI = 1.23–2.58), 3) glycochenodeoxycholic acid (DOR = 1.68: 95% CI = 1.13–2.48), 4) taurochenodeoxycholic acid (DOR = 1.62: 95% CI = 1.11–2.36), 5) glycohyocholic acid (DOR = 1.65: 95% CI = 1.13–2.40) and the secondary bile acids 6) glycodeoxycholic acid (DOR = 1.68: 95% CI = 1.12–2.54), and 7) taurodeoxycholic acid (DOR = 1.54: 95% CI = 1.02–2.31).^99^ Loftfield et al. also identified 7 bile acids strongly correlated with CRC risk including the primary bile acids 1) taurochenodeoxycholic acid (DOR = 2.98: 95% CI = 1.42–6.24) and 2) glycocholic acid (2.26: 95% CI = 1.21–4.22) as well as the secondary bile acids 3) deoxycholic acid (DOR = 2.85: 95% CI = 1.45–5.60), 4) glycodeoxycholic acid (DOR = 3.45: 95% CI = 1.79–6.64), 5) taurodeoxycholic acid (DOR = 2.36: 95% CI = 1.22–4.55) 6) glycolithocholic acid (DOR = 2.71: 95% CI = 1.41–5.22) and 7) taurolithocholic acid (DOR = 1.84: 95% CI = 1.16–2.93).^100^ These studies collectively indicate that bile acids, whether detected in the stool or serum, have the potential with further development to be utilized for early detection of colonic neoplasms.

## Short chain fatty acids

Short chain fatty acids (SCFAs) are small carboxylic acids 1–6 carbons in size that are produced through the fermentation of undigested dietary fibers by various groups of bacterial gut commensals, including members of the *Clostridium*, *Eubacterium*, and *Butyrivibrio* genera. Production of SCFAs is influenced by multiple environmental conditions of the gut including oxygen levels, pH, hydrogen sulfide, and the community composition’s competition for resources.^101,102^ The three most abundant and best characterized SCFAs are propionate, butyrate, and acetate, which collectively have numerous beneficial effects both within the colon as well on other organ systems.^103^ The well-characterized benefits that SCFAs confer to the gut include augmenting the barrier function of the intestinal epithelium, regulating intestinal stem cell turnover, increasing expression of intestinal epithelial tight junctions and intestinal epithelial oxygen consumption to maintain an anoxic environment.^102^ SCFAs also act on multiple immune cell populations including myeloid cells, T cells, B cells, and innate lymphoid cells to reduce gut inflammation, while maintaining protective antipathogen functions.^102,104^ The importance of SCFAs for modulating the immune system is underscored by the numerous autoimmunity conditions including multiple sclerosis, type I diabetes, graft versus host disease and allergies where SCFAs have either been observed at lower levels in these patients and/or SCFA-based interventions ameliorate disease.^102^

Studies comparing the levels of SCFAs in the stool of CRA or CRC patients and healthy controls have reported varying results. Several studies have found decreased levels of SCFAs in the stool of patients with CRA or CRC.^105–109^ However, there are also studies that have shown that SCFAs levels are higher in either CRA or CRC patients,^110,111^ while still other studies show that there are no differences between SCFA levels between cancer patients and healthy individuals.^112,113^ To collectively evaluate the large number of studies that have compared SCFAs between CRA or CRC patients and healthy controls and the variability in cohorts and results between these studies, a compilation of the results has been examined through a meta-analysis showing that butyrate, propionate, and acetate are at significantly lower levels in individuals with higher risk of CRC,^114^ supporting the model that SCFAs play a protective role against CRC tumorigenesis and the depletion of SCFAs being predictive of colonic lesion development.

Additional support for SCFAs being a capable biomarker for colonic lesions comes from the findings highlighted in the large cohort metagenomic, metabolomics, and functional pathways analyses highlighted in Tables 1–3. In Coker et al. butyric acid was the only metabolite significantly depleted in the fecal samples of CRC patients (*n* = 118) compared to both healthy controls (*n* = 128) and CRA patients (*n* = 140),^47^ while in Kong et al. acetate was significantly depleted in the stools of late onset colorectal cancer patients compared to those of their aged matched healthy controls.^42^ Furthermore, a consistent trend observed in varying combinations across these studies was the depletion of major SCFA producing taxa. Yachida et al. report significant depletion of butyrate producers *Lachnospira multipara* and *Eubacterium eligens* across all colonic lesion stages, while Liu et al. observed significant depletion of *Clostridium butyricum* and *Butyrivibrio fibrisolvens* in addition to *F. prausnitzii*, *Eubacterium eligens*, and *R. intestinalis*, which were among the bacterial taxa included in a 16 microbial feature panel for classification models that accurately differentiated CRC patients from healthy individuals with an AUC > 0.8 in the majority of validation cohorts.^58,59^ Likewise, both *F. prausnitzii* and *R. intestinalis* were both depleted species also included as features in classification models used by Gao et al. and Kong et al. for accurate healthy vs. CRC patient differentiation.^41,42^

**Table 2.** Bacterial functional pathways used for CRC detection.

Study	Feature Discovery and Validation Cohorts	Bacterial functional pathways used for classification	AUROC
Dai et al.^75^	4 publicly available CRC metagenomic datasets used for feature discovery and AUC measurement (healthy controls, *n* = 271; CRC cases, *n* = 255).	7 KEGG pathways enriched in CRC metagenomes including citrate cycle, LPS biosynthesis, ubiquinone and other terpenoid quinone biosynthesis, other glycan degradation, lipoic acid metabolism, valine/leucine/isoleucine degradation, phosphonate and phosphinate metabolism	Not evaluated
Wirbel et al.^57^	8 cohorts included − 1 new metagenomic dataset (healthy controls, *n* = 60; cancer cases, *n* = 60) and 4 additional publicly available CRC metagenomic datasets (healthy controls, *n* = 230; cancer cases, *n* = 225) used for feature discovery. Feature panel was evaluated in the 5 feature discovery datasets and 3 additional publicly available metagenomic CRC external cohorts (healthy controls, *n* = 92; cancer cases, *n* = 101)	Top gut metabolic modules, as defined in Vieira et al., enriched in CRC patients are amino acid degradation, organic acid metabolism, and glycoprotein degradation, while the top metabolic module depleted in CRC patients is carbohydrate degradation. qPCR quantified baiF levels in 47 stool samples for healthy vs. CRC patient classification.	Healthy vs. CRC classification AUC range of 0.78–0.89 by LOSO analysis across the 5 cohorts used for feature discovery and healthy vs. CRC classification AUC range 0.71–0.92 across the 3 external validation cohorts using classifiers based on eggNOG orthologous gene family abundances. Healthy vs. CRC classification AUC = 0.77 across 47 fecal samples using qPCR measured abundances of baiF.
Thomas et al.^61^	7 cohorts included − 2 new metagenomic dataset, cohort 1 (healthy controls, *n* = 24; CRA, *n* = 27; CRC, *n* = 29), cohort 2 (healthy controls, *n* = 28; CRC, *n* = 32) and 5 publicly available CRC metagenomic datasets (healthy controls, *n* = 256; CRA, *n* = 116; CRC, *n* = 252). Feature panel was evaluated for all 7 cohorts.	Significantly increased abundances of cutC, encoding choline TMA-lyase that is required for trimethylamine (TMA) synthesis, occur in CRC metagenomes compared to the metagenomes of healthy individuals.	Not evaluated
Yachida et al.^58^	Single new metagenomic dataset including a total of 616 people (healthy controls, *n* = 251; multiple polypoid adenomas with low grade dysplasia (MP), *n* = 67; stage 0, *n* = 73; stage I/II, *n* = 111; stage III/IV, *n* = 74; individuals with a history of colorectal surgery (HS) *n* = 40) used for feature discovery and AUC measurements.	KEGG pathway enriched in CRC metagenomes include aromatic amino acid metabolism and sulfide-producing pathways. 5 KO gene panel for AUC classification of healthy vs. SIII/IV CRC patients – features include *estA*, *kamD*, *aaC*, *ribG*, *doxD*. 16 KO gene panel for AUC classification of healthy vs. S0 CRC patients – features include pheC, K09961, *atpD*, TC.NCS1, *gltS*, *uidA*, *ycbB*, *fno*, *gcvPA*, *cheX*, *gnl*, *bdhAB*, *rmd*, K09960, *pel*, *ridA*	Healthy vs SIII/IV CRC classification AUC = 0.69 using a 5 KO gene panel. Healthy vs S0 CRC classification AUC = 0.75 using a 16 KO gene panel.
Liu et al.^49^	8 cohorts included − 1 new metagenomic dataset (healthy controls, *n* = 86; CRC, *n* = 80). Feature panel was identified with five publicly available CRC metagenomic datasets (healthy, *n* = 494; CRC, *n* = 491), Feature panel was evaluated in all 5 discovery cohorts and 3 additional metagenomic CRC datasets including the new metagenomic dataset (healthy, *n* = 190; CRC, *n* = 193).	KEGG pathways enriched in CRC metagenomes include carbohydrate metabolism (ie. butanoate, ascorbate, and aldarate), and D-arginine and D-ornithine metabolism. KEGG pathways depleted in CRC metagenomes include branched chain amino acid (valine, leucine, and isoleucine), and lipid metabolism. Significantly increased *bdhA*/*bdhB*, involved in butanoate metabolism, and *oraS*/*oraE*, involved in D-arginine and D-ornithine metabolism were qPCR validated as significantly enriched in CRC patient metagenomes. 175 eggNOG gene panel used for AUC classification of healthy individuals vs. CRC patients.	Healthy vs. CRC classification average AUC = 0.86 for the 5 cohorts used for feature discovery, using the 175 eggNOG gene panel.
Avuthu et al.^51^	3 publicly available CRC metagenomic datasets used for feature discovery. Feature panel was evaluated is the 3 discovery cohorts (healthy controls, *n* = 166; CRC, *n* = 180) and 2 additional publicly available CRC metagenomic datasets (healthy controls, *n* = 303; CRC, *n* = 304).	MelonnPan pathways enriched in CRC metagenomes include amino acid metabolism. Pathways depleted in CRC metagenomes include arginine biosynthesis, nicotinate and nicotinamide metabolism, D-glutamine and D-glutamate metabolism, and butanoate metabolism.	Not evaluated.
Kong et al.^42^	Total of 2 cohorts included − 1 new metagenomic dataset for feature discovery including a total of 441 people (healthy controls for late onset (LO) CRC (>50 years of age), *n* = 97; LO-CRC, *n* = 130; healthy controls for early onset (EO) CRC (<50 years of age), *n* = 100; EO-CRC, *n* = 114). One new metagenomic dataset for feature validation including a total of 108 people (healthy controls for LO-CRC, *n* = 22; LO-CRC, *n* = 38; healthy controls for EO-CRC, *n* = 24; EO-CRC, *n* = 24.	KEGG pathways related to amino acid metabolism urea cycle, including leucine/isoleucine biosynthesis, histidine biosynthesis, lysine biosynthesis are depleted in EO-CRC and LO-CRC patient metagenomes, while histidine degradation, leucine degradation, phenylalanine biosynthesis pathways are enriched in EO-CRC and LO-CRC patient metagenome. A 59 KO gene panel used for AUC classification of LO-CRC and LO-controls – top 10 KO gene features include *gltA*, *atpB*, *atpG*, *mcp*, *atpF*, *argD*, *atpD*, *atpC*, *speA*, and *leuA*. 59 KO gene panel used for AUC classification of EO-CRC and EO-controls – top 10 KO gene features include *birA*, *coaX*, *ycsE*, *speA*, *dacD*, *folC*, *glmM*, *mppA*, *gltA*, and *msmE*	LO-CRC vs. LO control AUC = 0.8211 using the 59 KO gene panel and EO-CRC vs. EO control AUC = 0.7552 using the 59 KO gene panel.

**Table 3.** Metabolites used for CRC detection.

Study	Feature Discovery and Validation Cohorts	Metabolites used for classification	AUROC	Sample Type
Yachida et al.^58^	Single new metabolomics dataset including a total of 406 people (healthy controls, *n* = 149; multiple polypoid adenomas with low grade dysplasia (MP), *n* = 45; stage 0, *n* = 30; stage I/II, *n* = 80; stage III/IV, *n* = 68; individuals with a history of colorectal surgery (HS) *n* = 34) used for feature discovery and AUC measurements.	65 metabolites significantly enriched or depleted in CRC patients compared to healthy controls. Deoxycholate, glycocholate, and taurocholate were found at higher concentrations in individuals with early stage colonic lesions (MP and S0) compared to healthy controls. Branched chain amino acids (isoleucine, leucine, and valine), phenylalanine, tyrosine, and serine were increased in varying CRC stages. 62 bacterial metabolite panel used for AUC classification of healthy vs SIII/IV CRC patients – top features include urocanate, N,N-dimethylglycine, hydroxyproline, serine, 5-hydroxylysine, and N-acetylglucosamine 1 phosphate. 24 bacterial metabolite panel used for AUC classification of healthy vs S0 CRC patients – top features include leucine, valine, phenylalaine, lactate and succinate.	Healthy vs SIII/IV CRC classification AUC = 0.74 using a 62 bacterial metabolite panel. Healthy vs S0 CRC classification AUC = 0.65 using a 24 bacterial metabolite species panel.	Stool
Gao et al.^41^	2 cohorts included − 1 metabolomics dataset (healthy controls, *n* = 34; CRC, *n* = 31; CRA, *n* = 35) used for feature discovery. Feature panel was evaluated in 1 additional CRC metabolomics dataset (healthy, *n* = 76; CRC, *n* = 113)	8 metabolic pathways significantly enriched or depleted in CRA or CRC patients compared to healthy controls, including 29 metabolites (including 13 amino acids), differentially abundant between CRA patients and healthy controls, and 59 metabolites (including 22 amino acids) between CRC patients and healthy controls. CRA enriched metabolites included N-methylproline, trigonelline, and CRA depleted metabolites included sphingosine and sphinganine. CRC enriched metabolites include leucylglycine, N-acetylneuraminate, isoleucylglycine, phenylacetylcarnitine, and serotonin, and CRA depleted metabolites include 3-hydroxylaurate, perfluorooctane sulfonate, and uracil. 3 bacterial metabolite panel, including N(1)+N(8)-acetylspermidine, 2-linoleoylglycerol(18:2), perfluorooctane sulfonate used for AUC classification of healthy vs CRC patients	Healthy vs. CRC classification AUC range of 0.821 (95% CI:0.761–0.882) in the validation cohort using the 2 bacterial metabolite panel.	Blood serum
Coker et al.^47^	One new metabolomics dataset including a total of 386 people used for feature discovery and AUC measurement (healthy controls, *n* = 128; CRA, *n* = 140; CRC, *n* = 118)	17 metabolites significantly enriched or depleted in CRC patients. The CRC enriched metabolites compared to healthy controls include L-alanine, glycine, L-proline, L-aspartic acid, L-valine, L-leucine, L-serine, myristic acid, phenyl lactic acid, oxoglutaric acid, L-phenylalanine, L-alpha-aminobutyric acid, phenylacetic acid, palmitoleic acid, 3-aminoisobutanoic acid and norvaline. The top 4 enriched pathways in CRC compared to healthy controls 1) aminoacyl-tRNA biosynthesis 2) valine, leucine, and isoleucine biosynthesis 3) phenylalanine metabolism 4) phenylalanine, tyrosine and tryptophan biosynthesis. 20 or 11 bacterial metabolite panels were used for AUC classifications of healthy vs CRC patients.	Healthy vs. CRC classification AUC = 0.8, CRC vs CRA classification AUC = 0.7889 using a 20 bacterial metabolite panel. Healthy vs CRA classification AUC = 0.6853, and CRC vs CRA classification AUC = 0.7464 using an 11 bacterial metabolite panel. CRC vs CRA classification AUC = 0.81, CRC vs. healthy controls AUC = 0.7168, and CRA vs. healthy controls classification AUC = 0.6648 using a 13 bacterial metabolite panel.	Stool
Kong et al.^42^	Total of 2 cohorts included − 1 new metabolomics dataset for feature discovery including a total of 441 people (healthy controls for late onset (LO) CRC (>50 years of age), *n* = 97; LO-CRC, *n* = 130; healthy controls for early onset (EO) CRC (<50 years of age), *n* = 100; EO-CRC, *n* = 114). One new metabolomics dataset for feature validation including a total of 108 people (healthy controls for LO-CRC, *n* = 22; LO-CRC, *n* = 38; healthy controls for EO-CRC, *n* = 24; EO-CRC, *n* = 24.	162 differentially abundant metabolites between LO-controls and LO-CRC, and 167 differentially abundant metabolites between EO-controls and EO-CRC. LO-CRC depleted metabolites relative to LO-controls include acetate, acetaldehyde, L-arginine and LO-CRC enriched metabolites include perfluorooctanesulfonic acid, D-ornithine, and L-phenylalanine. The EO-CRC enriched metabolites relative to EO-controls include perfluorooctanesulfonic acid, indole-3-actylaldehyde, L-phenylalaine, L-aspartate, glycine, tryptophan, D-ornithine, 1-acyl-sn-glycerol-3-phosphocholine, phosphatidylcholine, linoleate, choline, and deoxycholic acid. 16 bacterial metabolite panel used for AUC classification of LO-control vs LO-CRC classification in the validation cohort. 36 bacterial metabolite panel used for AUC classification of EO-control vs EO-CRC classification in the validation cohort.	LO-control vs LO-CRC classification AUC = 0.7847 in the validation cohort using 16 bacterial metabolite panel. EO-control vs EO-CRC classification AUC = 0.7535 in the validation cohort using the 36 bacterial metabolite panel.	Stool

Additionally, a study comparing butyric acid levels in the serum between healthy individuals (*n* = 20), CRA patients (*n* = 26), CRC patients (*n* = 19), and familial adenomatous polyposis patients (*n* = 10) found that butyric acid was one of the top distinguishing features at increased abundance in CRA patients compared to healthy controls.^110^ It is clear that SCFA impacts CRC tumorigenesis through multiple mechanisms; however, the use of SCFAs to reliably differentiate CRA or CRC patients from healthy individuals remains uncertain given the discrepancies across studies as to whether SCFAs are elevated or depleted in CRA or CRC patients and further work will be required to discern the utility of SCFA abundances for colonic lesion detection.

## Polyamines

Polyamines are small polycationic alkylamines including putrescine, spermidine, and spermine that are acquired through diet while also being produced and taken up by rapidly proliferating cells such as enterocytes. Microbial commensals also produce polyamines through the fermentation of arginine.^115,116^ Polyamines are intricately linked to multiple types of cancer with mutations in several oncogenes including *MYC*, *RAS*, *PTEN*, and *WNT* resulting in increased production, uptake, and utilization of polyamines required for tumor proliferation.^117,118^ The role that polyamines play in CRC proliferation has led to the development of treatment strategies, such as difluoromethylornithine paired with non-steroidal anti-inflammatory agents, that target polyamine synthesis.^119^ The well-documented role of polyamines in CRC tumorigenesis has motivated efforts to measure polyamines in urine, stool and serum as a diagnostic for CRC. Seven polyamines profiled in the urine from a cohort of 201 CRC patients and 31 healthy individuals showed that N1,N2-diacetylspermidine alone could classify CRC cases from healthy individuals with an AUC = 0.794 (95% CI = 0.704–0.885) and a combination panel of polyamines was able to discriminate patients from healthy controls with an impressive AUC = 0.961 (95% CI: 0.937–0.984)^120^ The ability to detect whether individuals have colonic lesions through measuring polyamine levels in the urine is supported by two additional studies reporting similar findings that polyamines levels are elevated in CRC patients.^121,122^ Within stool, levels of N-acetyl putrescine and cadaverine were detected at 2 to 25-fold higher abundances from individuals with precancerous lesions/adenocarcinomas compared to healthy individuals.^123^ An even greater differential abundance in polyamines was observed comparing the serum of CRC patients and healthy individuals, most notably spermidine and acetyl putrescine that were detected at approximately 140-fold and 960-fold higher abundances among a cohort of 50 CRC patients and 52 healthy controls.^124^ Collectively, these studies indicate that polyamines are a viable prognostic for CRC warranting further investigation and development.

## Trimethylamine-N-oxide

Trimethylamine-N-oxide (TMAO) is an oxidized form of trimethylamine that is produced from precursor choline and L-carnitine acquired from the diet from items such as eggs, red meats, and milk. Choline is converted to trimethylamine by gut commensals encoding the choline lyase (*cutC*), while L-carnitine is converted to trimethylamine primarily by the *bbu*/*gbu* gene clusters, however, the *cntA* pathway is utilized by some microbes under aerobic conditions.^125–128^ Trimethylamine is then oxidized to TMAO by hepatic flavin monoxygenase in the liver.^129^ TMAO can also be acquired directly from consumption of seafood.^130^ The link between CRC and TMAO was first reported in a study that found a positive correlation between serum levels of TMAO and higher risk of CRC.^131^ This initial finding suggesting TMAO is associated with increased risk of CRC, has since been further supported by additional studies that have reported elevated levels of TMAO in the serum of CRC patients compared to healthy controls.^132,133^ Further support for TMAO contributing to CRC was provided by Xu et al. with epigenetic interaction networks and genome wide association methods showing that TMAO is strongly associated with CRC.^134^ However, one study has shown that TMAO levels are not elevated among CRC patients, but rather the TMAO precursor, choline, was elevated among CRC patients.^135^ Unlike bile acids and SCFAs, where many different protective or tumorigenic molecular mechanisms have been described, much less is known as to how TMAO or TMAO precursors promote CRC. Mechanisms that have been described include TMAO driven inflammation through the NF-kappa-B signaling, and ROS mediated NLRP3 activation.^136,137^

In addition to elevated serum levels of either TMAO or choline, metabolic and metagenomic profiling of CRC patient stool samples has indicated that markers related to choline metabolism could be potentially used for a CRC diagnostic. Thomas et al. reported significantly increased abundance of the bacterial *cutC* gene in the pooled CRC metagenomes compared to healthy controls and choline was detected at significantly higher levels in the stools of early onset CRC patients in the metabolomics profiling done in Kong et al.^41,42,57^

Both the molecular mechanisms of TMAO and TMAO precursor metabolites driven CRC tumorigenesis and the correlation between these molecules and CRC risk will require further work. However, the work highlighted here indicates that TMAO measurements in the serum or stool could be leveraged for an early detection diagnostic for colonic lesions.

## Indoles

Indole’s role in the microbiome and in CRC is particularly complex, with both pro-tumorigenic and tumor suppressive mechanisms observed. Indole plays an extensive role in microbial signaling and has even been implicated as an interkingdom signaling molecule.^138,139^ Indole can influence bacterial growth and pathways including spore formation, biofilm formation, alkaline response systems, and even plasmid stability.^138^ Evidence that indoles play a protective role against CRC includes the observation that indoles are depleted in the stool of CRC patients and that indole derivatives such as indole-3-carbinol have been shown to inhibit CRC progression.^140,141^ Furthermore, microbially derived indoles are able to have an impact on CRC development and progress, where indole-3-lactic acid (ILA) produced by *Lactobacillus pantarum* can impede tumorigenesis via regulation of the host immune system.^142^ While ILA on its own can impede tumorigenesis, additional work has shown that that *Lactobacillus* mediated production of ILA leads to increased expression of metabolic pathways involved in the production of additional indole metabolites such as indole-3-propionic acid and indole-3-acetic acid, which have also been implicated in repressing intestinal inflammation.^139^ Additionally, indole can repress the growth of bacteria associated with the development of CRC including *E. coli* .^143^ However, indole metabolism remains a multifaceted component of CRC as it has also been shown to induce biofilm formation in *F. nucleatum*, which may affect its role in mediating CRC progression.^144,145^ Indole’s utility as a diagnostic marker of CRC remains underexplored; however, in conjugation with multi-omic approaches it may prove to be effective for monitoring tumorigenesis.

## Branched chain amino acids and phenylalanine

Alterations in amino acid metabolism-related functional pathways were observed in several of the studies highlighted in Table 2. These altered functional pathways include both biosynthesis and degradation of several amino acids, which is consistent with the growing body of work elucidating how amino acids are utilized by different cancer types, which has been well reviewed elsewhere.^146,147^ However, two functional pathways that were consistently observed as most enriched in stool-derived CRC-associated metagenomes include branch chain amino (BCAA) (valine, leucine, and isoleucine) and phenylalanine biosynthesis pathways. For instance, *pheC*, which encodes cyclohexadienyl hydratase involved in phenylalanine biosynthesis, was the highest contributing feature to predictive models in Yachida et al. that included bacterial taxa, metabolites, and functional pathways for differentiating S0 CRC patients from healthy controls.^58^ Likewise, pathway analysis based on the stool metabolomics analysis in Coker et al. identified BCAA biosynthesis, phenylalanine metabolism, and phenylalanine, tyrosine, and tryptophan biosynthesis as three of the top four enriched pathways of CRC metabolomes relative to that of healthy individuals and CRA patients.^47^ The involvement of these pathways in CRC tumorigenesis is further supported by the metabolomics findings from these studies. Yachida et al. observed significantly increased levels of all three BCAAs in at least one of the four stages of colonic lesions included in their study, while isoleucine was among a of group amino acid metabolites enriched across both CRA and CRC metabolomes in the Gao et al. metabolomics profiles.^41^ In Kong et al., only two metabolites, including phenylalanine, were significantly enriched in both early-onset CRC and late-onset CRC compared to their age matched controls, while functional pathway analysis in this study showed that phenylalanine biosynthesis genes *aroA* and *phe*A2 were enriched in the metagenomes of all CRC patients relative to controls.^41,42^

In addition to the studies highlighted in Tables 2,3, other studies exploring metabolites enriched in patients with colorectal lesions have identified associations between phenylalanine or BCAAs and CRC. An eight-feature panel of metabolites significantly elevated in the stool samples collected from CRA and CRC patients, including phenylalanine (N,O-Bis-trimethylsily(phenylalanine)), achieved an AUC = 0.92 and 0.84 for differentiation of CRC vs healthy and CRA vs healthy control, respectively, in the validation cohort.^40^ Another study investigating functional pathways enriched in CRC or CRA based on stool metabolite profiles identified phenylalanine, tyrosine, and tryptophan metabolism and phenylanine metabolism as two of the four pathways most enriched in CRC patient stool metabolomes.^86^ Analyses of CRC or CRA patient serums have also indicated that systemically circulating phenylalanine and other amino acids are differentially abundant and can potentially be used for colonic tumor detection.^148,149^

Tumor amino acid metabolism is complex with our understanding of how tumors utilize various amino acids for numerous functions continuing to grow. The role that phenylalanine and the BCAAs play in CRC in not yet fully understood; however the metagenomic, metabolomic, and functional pathway analyses from these datasets suggest that the altered levels of these amino acids could be potentially incorporated into a future diagnostic for detecting colonic lesions.

## Combining CRC-associated metabolite markers for improved diagnostics

The metabolite markers described above include several of the best characterized markers for indicating the presence of colonic lesions and potential lead candidates for metabolite-based CRC/CRA diagnostics. The variety of these markers and the multiple biospecimens that these metabolites have been evaluated in indicate there is an opportunity to potentially improve the accuracy of metabolite-based classifiers through approaches that combine measurements of several metabolites. Work to evaluate the classification accuracy of metabolite marker combinations, including those discussed in depth above, has begun as detailed in the studies included in Table 3. In these studies, the classification accuracy for differentiating healthy individuals from CRA/CRC patients with differing stages of disease or timing of disease onset using combinations of metabolites measured in biospecimens including serum, blood, and stool were evaluated. Collectively, these studies indicate that metabolite markers have varying predictive value with similar limitations for differentiation of healthy from disease-associated individuals as those observed in efforts to use bacterial taxa-based panels. Namely the most accurate classification occurs between CRC, especially later-stage disease, versus CRA or healthy individuals, as compared to classification between CRA and healthy individuals.^47,58^ Additionally, variability between the composition of the panels used for classification between studies indicates a potentially large breadth of markers that contribute to classification accuracy with the predictive value of the markers dependent on the study cohort and the biospecimen from which they are measured.

Despite these challenges, continued investigation of metabolites associated with progression along the adenoma-carcinoma sequence has further refined which metabolites provide the best diagnostic value and how metabolite signatures derived from patient serum or stool compare in their classification accuracy. In Sun et al., stool and serum collected from a large discovery cohort of 91 CRC and 115 CRA patients and 109 healthy controls were analyzed, and a panel of 17 metabolites providing the best discrimination between CRC and healthy donor samples, including 7 CRC enriched and 10 CRC depleted metabolites, was evaluated for classification accuracy in three validation cohorts.^150^ Impressively, the 17 serum-derived metabolite profile provided AUC values greater than or equal to 0.85 for discriminating between healthy controls and both late- and early-stage CRC, as well as CRA versus CRC in all three validation cohorts. A stool-derived panel of 19 metabolites was also evaluated and compared to the serum-based panel and was shown to provide comparable but overall lower classification accuracy compared to the 17 serum-derived metabolite panel. Notably, this study evaluated the classification accuracy of the combined serum- and stool-derived metabolite panels, finding that addition of the stool-derived metabolites did not significantly improve the classification accuracy of the 17 serum-derived metabolite panel. These important findings suggest that metabolite signatures from easily collected serum samples are sufficient for reasonably accurate detection and differentiation of early and late CRC-associated colonic lesions and provide a roadmap for further investigation and diagnostic development of the metabolites with the highest potential for diagnostic utility in CRC patients.

## Challenges of microbiome and metabolite-based CRC diagnostics

As progress toward defining a clear CRA/CRC-associated gut bacterial signature continues, it is important to recognize some of the challenges that remain. One of these challenges and a consideration for any effort to assign a specific microbiome signature to a human pathology is identifying and controlling for the numerous potential covariates responsible for microbiome variability across individuals and parsing microbiome variability due to one or a combination of these covariates from the microbiome variability associated with disease. Our understanding of covariates that impact microbiome variability in healthy individuals has improved through the studies that have integrated microbiome datasets with comprehensive clinical and questionnaire-based metadata from large human cohorts.^151–154^ These studies have shown that covariates responsible for the largest interindividual microbiome variability include Bristol stool score, which is a measurement of stool consistency, age, body mass index (BMI), various components of the diet including alcohol, coffee, and fiber consumption, smoking, inflammatory bowel conditions, medication, and the geographical location of the individual, among others.^151–154^ In addition to these large cohort studies integrating microbiome and metadata datasets to identify covariates impacting microbiome variance, some specific covariates such as diet, and the use of certain medications such as antibiotics have been investigated in depth and detailed insights into how these covariates impact microbiome variance have been described.^155–161^ Despite the growing understanding of these covariates and their importance to inter-individual microbiome variance, studies attempting to attribute microbiome signatures to pathologies rarely control for these confounding variables leaving open the possibility that the associations reported between a disease and the microbiome signature are occurring due to a combination of confounding covariates and the disease. Indeed, the importance of fully matching covariate controls between disease populations and “healthy” controls was demonstrated in Vujkovic-Cvijin et al. where beta-diversity between disease associated, including cancer, and “healthy” control stool-derived microbiomes shrank considerably when confounding covariates where matched between the two populations.^153^ Despite these challenges, progress has been made toward defining a CRC-associated gut microbiome and disentangling microbiome variability driven by the CRA or CRC diagnosis from covariates, as well as identifying covariates that do not impact consistent CRC-associated microbiome signatures. For instance, Tito et al. included rigorous covariate control focusing on fecal moisture content, calprotectin, a marker for bowel inflammation, and BMI as covariates for microbial diversity. These covariates explain more microbiome variance than diagnostic classification between CRA/CRC patients and healthy control populations from data collected from multiple studies, demonstrating the importance of controlling for these covariates while also pointing to potential molecular and clinical markers that can be paired with microbiome profiling to improve colonic lesion detection.^54^ Conversely, it is equally important to identify covariates that seem to have minimal impact on interindividual stool microbiome variance of CRA/CRC patients. In Young et al. the impact of geography and the well-documented variance in the microbiome of individuals from industrialized and developing countries on CRA/CRC microbiome signatures was evaluated, indicating that the CRC-associated microbiome is consistent between individuals in industrialized and developing countries.^162^ As recognition and control for important confounding covariates improves, the CRA/CRC-associated microbiome signature will continue to be refined and better defined.

Another challenge to identifying CRC or other disease-associated microbiome signatures stems from the reliance on relative abundance measurements, a commonly used metric in the microbiome field. While relative abundance measurements are a powerful tool, enabling rapid profiling of the GM taxonomic and functional composition, they are subject to distortions. One such distortion is when an overall depletion of the entire microbiome is occurring, but some taxa are spared from depletion and remain at the same abundance. In this case, relative abundance measurements would inaccurately indicate an increased abundance of the taxa spared from depletion. To overcome the limitations of relative abundances, some studies have started collecting absolute abundance measurements, which is done by ‘spiking-in’ a known quantity of DNA from an organism not found in the microbiome being investigated and normalizing the abundance measurements of the community taxa to the ‘spike-in’ DNA (Figure 2(b)). Absolute abundance measurements allow for more accurate quantification of microbial loads and the directionality of changes in overall microbial communities and taxa of interest as demonstrated in Vandeputte et al. and are becoming more widely adopted.^163^ As the use of absolute abundance measurements grows and becomes standard practice, as has been done in Tito et al. in their work investigating the CRC-associated microbiome, understanding of the taxa enriched and depleted during CRC tumorigenesis will continue to improve.^54^

Meanwhile, metabolomics studies have noted that variation in sample source results in varied performance and metabolites associated with CRC (i.e. urine, plasma, tumor tissue, and stool).^109,164,165^ Systematic and summary reviews have compared the metabolites identified from these different sample sources and performance across different studies. Currently, it remains unclear if certain sample types exhibit greater diagnostic utility in part due to the small sample sizes of studies, the outcome studied (i.e. CRC or polyps), and disagreements among studies. Notably, Seum et al. collected 26 studies for a systematic review focusing on early detection of CRC, 12 of the studies reported AUCs (6 studies utilizing plasma/serum, 3 studies utilizing stool, 3 urine studies). Stool studies reported the highest AUCs (AUC of 0.94,^166^ 0.95,^167^ 0.97)^86^ followed by plasma/serum studies (AUC of 0.76,^168^ 0.82,^169^ 0.83,^170^ 0.85,^170^ 0.87,^170^ 1.0)^171^ and urine studies reported the consistently lowest AUCs (AUC of 0.69,^172^ 0.72,^173^ 0.72)^174^ However, other stool and blood studies we discussed in this review reported more modest AUCs (Table 3). Variability in sample type performance may depend on two factors: 1) studies utilizing smaller cohorts 2) impact of lifestyle choices. Firstly, small cohorts might fail to capture subtle shifts in biomarkers and model performance on smaller cohorts is often artificially inflated.^164,165^ Secondly, urine and stool sampling is subject to strong dietary influences, which can even impact the GM.^175,176^ While blood and tissue sampling approaches are less susceptible to dietary shifts but are more invasive, which may limit the feasibility of repeated tissue sampling. The ease of access and preliminary indications of higher accuracy for CRC detection from stool-derived metabolites point toward stool being a promising biospecimen for metabolomics-based diagnostics.

Other challenges to the implementation of microbiome-based diagnostics arise from the collective logistical considerations that range from uniform sample collection and storage, reducing contamination that can occur at multiple points during sample collection and processing, the computational tools and expertise required for data analysis, the standardization of analyses to allow clinicians to interpret and utilize the data, the costs associated with each of these steps, and the regulatory hurdles that must be cleared for implementing novel diagnostics into the clinic. Detailed overviews of these challenges and the various strategies and innovations that have been developed to overcome these challenges have been published elsewhere.^177–179^ These reviews, among others, highlight progress that has occurred on multiple fronts, including the steps that occur before a sample is included in a microbiome sequencing experiment, to best practices for analyzing, and sharing microbiome data. For instance, the publication of CEN/TS 17,626:2021, ‘Molecular in vitro diagnostic examinations-Specifications for pre-examination processes for human specimen-Isolated microbiome DNA’, which is a European pre-analytical standard for human specimens intended for microbiome DNA analysis that provides requirements and gives recommendations regarding specimen collection, handling, storage, processing, DNA isolation, and documentation for microbiome studies is an important step toward standardizing the critical steps prior to including a sample in a microbiome sequencing experiment.^180^ In addition to standardizing requirements for the pre-analytical steps, efforts are being made to develop best practices for data deposition to improve accessibility, integration, and reuse of microbiome data.^178^ Notable developments include the implementation of checklists such as MIxS and STORM, that allow tracking and sharing of sample collection methods, sample properties, nucleotide extraction method, sequencing methods, among other details to better document and link metadata to the samples included in microbiome experiments.^181^ Adoption of such standardized practices is key for microbiome-based approaches to be incorporated into CRC screening.

## Augmenting conventional diagnostic tools

Early works evaluating the diagnostic utility of microbial features tended to report lower performance than conventional diagnostic approaches.^182^ While work adjusting for interpatient variability and the adoption of more quantitative sequencing approaches will likely lead to improvements in future microbiome focused approaches,^54,163^ studies have also found that augmenting conventional diagnostic tools such as FIT with microbiome features can also improve the diagnostic performance.^55,183^ Notably, Baxter et al. and Wong et al. both demonstrated that integrating microbiome markers with FIT tests showed improved diagnostic utility.^55,183^ Baxter et al. observed that while microbial features were less effective for diagnosing CRC than conventional FIT tests, microbial features enabled the detection of lesions missed by FIT. Furthermore, incorporating these features into a single model resulted in significantly improved sensitivity capturing more instances of cancer and lesions at the expense of an increased false-positive rate.^183^ Wong et al. noted that using quantitative methods to derive microbial features significantly increased the sensitivity and AUC for detecting CRC and advanced adenomas. Importantly, these results could be recapitulated in validation cohorts as well.^55^ Interestingly, the potential for microbial features to augment existing diagnostic approaches has been shown to extend to MT-sDNA-based approaches improving the diagnostic accuracy when predicting CRC.^184^ Given that some tests such as FIT require the same source material as stool-based microbial tests, it is plausible that multimodal screening assessments may compensate for the shortcomings of different approaches.

## Conclusion

Screening and diagnosis of CRC depend on fecal or guaiac tests and colonoscopies respectively; however, these approaches are not sufficient for capturing microbiome-mediated risk factors such as the enrichment of risk-associated taxa or the depletion of protective pathobionts. These approaches have improved CRC detection, and lowered the morbidity and mortality of CRC, however there is room for improvement, particularly for earlier detection and more frequent testing, through more accessible diagnostics. We propose in this review that given the critical role of the microbiome and associated metabolites in modulating CRC onset and progression it is becoming increasingly apparent that evaluating the microbiome composition, function, and by-products produced from a CRC-associated microbiome may provide noninvasive diagnostic insights. These insights range from identifying elevated risk factors of CRC, and potential mediators of CRC progression to augmenting currently used diagnostic tools. Importantly, sequencing costs and the need for sequencing centers have traditionally been a major roadblock for utilizing microbiome markers, especially in areas without access to such facilities. While metabolomics approaches still depend on specialists and costly, large equipment, sequencing-based approaches have recently become much more affordable and the development of in-house approaches such as those produced by Oxford Nanopore Technologies has made sequencing much more cost effective and accessible.^8,185^ Unfortunately, standardized approaches and methods that compensate for interpatient variability need to become more widespread to ensure the clinical adoption of microbial markers.^37,186^ Nonetheless, given the ongoing research identifying diagnostic utility of microbial markers and improved accessibility of sequencing, we envision a future where microbiome-based markers may contribute to monitoring CRC risk and CRC diagnosis.

## Data Availability

Data sharing is not applicable to this article as no new data were created or analyzed in this study.
